# Family Outbreaks of Nontyphoidal Salmonellosis following a Meal of Guinea Pigs

**DOI:** 10.1155/2015/864640

**Published:** 2015-03-02

**Authors:** John B. Fournier, Kimberly Knox, Maureen Harris, Michael Newstein

**Affiliations:** ^1^Boston University School of Medicine, USA; ^2^Inpatient Dermatology Consultative Service, Roger Williams Medical Center, Providence, RI 02908, USA; ^3^Milford Regional Medical Center, Milford, MA, USA; ^4^University of Massachusetts Medical School, 55 North Lake Avenue, Worcester, MA, USA

## Abstract

*Salmonella* outbreaks have been linked to a wide variety of foods, including recent nationwide outbreaks. Guinea pig (*Cavia porcellus*), also known as cuy or cobayo, has long been a popular delicacy and ceremonial food in the Andean region in South America. This case report describes three family outbreaks of nontyphoidal salmonellosis, each occurring after a meal of guinea pigs. We believe this case report is the first to describe a probable association between the consumption of guinea pig meat and human salmonellosis. Physicians should be aware of the association of *Salmonella* and the consumption of guinea pigs, given the increasing immigration of people from the Andean region of South America and the increasing travel to this region.

## 1. Introduction

Salmonellosis continues to be a significant public health issue in the United States. The incidence of nontyphoidal* Salmonella* infections is estimated at 1 million annually in the United States and is responsible for 400 deaths each year [1]. Despite such estimates, the number of confirmed cases annually is considerably lower (~40,000), which is likely the result of milder cases going undiagnosed [2]. Symptoms of salmonellosis typically develop 6–72 hours following exposure and often include diarrhea, abdominal pain, and fever. In the majority of patients infection is self-limited, although infection can result in more serious symptoms and in some cases death, particularly in young children, the elderly, and immunocompromised patients.

Although few documented cases of salmonellosis have been linked to contact with guinea pigs,* Salmonella* is commonly isolated from guinea pigs and is associated with a wide range of morbidity and mortality in animal colonies [3]. The incidence of salmonellosis in guinea pigs approaches 25% [4–8]. In guinea pig laboratory colonies,* Salmonella* Typhimurium is the most common serotype, with* S*. Enteritidis,* S*. Weltevreden, and* S*. Poona being other commonly occurring serotypes [9, 10]. Regardless of serotype, infected guinea pigs often experience gastrointestinal symptoms, and* Salmonella* may disseminate to the heart, lungs, and kidneys [4]. Guinea pigs that recover from infection serve as latent carriers and are capable of transmitting* Salmonella *to other animals and humans.

One possible means of* Salmonella *transmission to humans is through the consumption of guinea pig meat, known as cuy in the Andean regions of South America. Cuy is a traditional delicacy in the Andean regions of South America, and the variety of preparation styles date back to pre-Columbian times [11]. Cuy is typically cooked and washed and can be prepared whole or cut into small pieces. The skin and organs of the animal are then removed with a knife. A marinade of onions, garlic, and black pepper is often applied, and the cuy is typically cooked for 30–60 minutes, often on hot stones known as pachamanca [11]. Although cuy has long been popular in South America, it has gradually been gaining popularity in the United States over the last several decades [12]. During this time period, shipments of frozen guinea pigs to the United States have increased considerably, with Peru's two largest commercial exporters shipping approximately 20,000 guinea pigs a year to the United States [12]. Assuming that increased immigration from Ecuador and Peru is at least partially responsible for the greater demand of cuy, and if current immigration trends continue, then guinea pig imports to the United States may continue to rise.

## 2. Case Reports

From 2006 to 2009, there were seven confirmed cases of* Salmonella *infections following the consumption of cuy, at Milford Regional Medical Center, in Milford, Massachusetts. Five of these cases occurred in Ecuadorian individuals who shared a meal of cuy in 2006. These five individuals consumed guinea pigs that were cooked in Ecuador, then frozen and wrapped in layers of metal foil, and shipped to the United States in a cardboard box without refrigeration. Within 6–24 hours after the meal, those individuals who had eaten cuy developed abdominal pain and diarrhea, with two individuals reporting bloody diarrhea. These five individuals were evaluated in the emergency department and other presenting symptoms included fever, nausea, and vomiting. Radiographic findings in three individuals revealed right lower quadrant fat stranding, suggestive of an inflammatory process. Stool cultures were obtained and* Salmonella* Group B: S.I 4,12:i:- was identified in all five samples. The individuals were admitted and received supportive treatment. Symptoms had either completely resolved or improved significantly within 2-3 days of admission; all individuals were able to tolerate oral nutrition by the third day of hospitalization and were subsequently discharged. It is of note that six other individuals were present at this meal of guinea pigs, and although five of these individuals also developed similar symptoms they did not seek medical attention. The lone individual who ate a vegetarian meal did not become ill.

In addition to the individuals who became ill in 2006, two other cases of confirmed* Salmonella* infections occurred following consumption of cuy. In 2008, a recent immigrant from Ecuador ate cuy that was shipped to the United States in a cardboard box. Reportedly, the box did not contain dry ice or another refrigeration source. Within 24 hours of eating cuy, the individual developed diffuse abdominal pain, nonbloody diarrhea, nausea, vomiting, and fever. The organism identified in this case was* S*. Typhimurium and the patient improved within two days of receiving supportive treatment. Two other family members attended this meal and developed similar symptoms, although their symptoms reportedly improved more quickly, and they did not seek medical treatment.

The most recent case of salmonellosis occurred in 2009 and involved an Ecuadorian immigrant who developed GI symptoms 12–24 hours after eating cuy. Like the other cases, this individual developed abdominal pain, diarrhea, nausea, and vomiting. Stool cultures were obtained in the emergency department and* S*. Typhimurium O:5- was identified. This patient received supportive treatment and symptoms resolved within three days. Reportedly, one other family member also ate cuy with this patient and developed similar symptoms. It is of note that, of the seven confirmed cases of salmonellosis, no patients developed leukocytosis, although several patients had band forms visualized on blood smears on initial presentation to the emergency department. Two of the seven patients also complained of muscle aches on presentation, although these symptoms improved during the course of the hospitalization.

## 3. Discussion

These cases of salmonellosis emphasize the importance of proper food storage and preparation. It is of note that the individuals who developed salmonellosis consumed cuy that was prepared and shipped to the United States by relatives in Ecuador and not prepared commercially. These guinea pigs were shipped from Ecuador to the United States without refrigeration, which likely provided an environment conducive to bacterial growth.

Additionally, these cases suggest that many individuals with salmonellosis are not receiving medical attention. Since the practice of shipping guinea pigs to the United States is common, it is likely then that many cases of salmonellosis are undiagnosed.* Salmonella* infections also may be underreported because of the failure to obtain a dietary history or to collect a stool sample for culture in individuals presenting with gastrointestinal complaints.

These outbreaks of salmonellosis are the first documented cases of infection in humans that result from eating cuy. Many guinea pigs shipped to the United States originate in specialized farms in Ecuador and Peru. Such farms house hundreds of guinea pigs at one time, and the close proximity of animals may increase the likelihood of* Salmonella *colonization. With virtually all guinea pigs shipped to the United States originating in Peru and Ecuador, the possibility exists for widespread dissemination of* Salmonella* throughout the United States. It is of note, however, that the cuy implicated in these three outbreaks of* Salmonella* was prepared by family members in Ecuador and shipped through the mail, rather than commercially prepared and shipped.

## 4. Conclusion

Immigration from Peru and Ecuador has risen considerably over the last several decades and this suggests that the demand and consumption of guinea pigs in the United States may continue to rise [13]. In areas with large populations of immigrants from Ecuador and Peru, medical personnel should be aware of the probable association between eating guinea pigs and salmonellosis. Furthermore, medical and public health professionals ideally should focus on patient education, with an emphasis on discussing proper shipping techniques for those individuals who wish to continue the practice of shipping guinea pigs. More specifically, individuals should be informed that guinea pigs/cuy should be shipped with dry ice or with a substitute, which allows for the meat to remain cold throughout transit.
